# Editorial: New threats of antibiotic-resistant bacteria and fungi

**DOI:** 10.3389/fmed.2022.1078940

**Published:** 2022-11-18

**Authors:** Shifeng Huang, Yi-Wei Tang, Christina A. Cuomo, Hui Wang, Xiaojiong Jia

**Affiliations:** ^1^Department of Laboratory Medicine, The First Affiliated Hospital of Chongqing Medical University, Chongqing, China; ^2^Medical Affairs, Danaher Diagnostic Platform China/Cepheid, Shanghai, China; ^3^Infectious Disease and Microbiome Program, Broad Institute of MIT and Harvard, Cambridge, MA, United States; ^4^Department of Laboratory Medicine, Peking University People's Hospital, Beijing, China; ^5^Harvard Medical School, Boston, MA, United States

**Keywords:** antibiotic resistance (ABR), multidrug resistance, CRE, bacterial resistance, fungal resistance

Today, antimicrobial resistance (AMR) continues to pose a major threat to our community and healthcare systems. It is estimated that AMR will cause 10 million deaths worldwide by 2050, surpassing cancer deaths. Multidrug-resistant (MDR) pathogens which are frequently resistant to almost all antibiotics significantly contributes to the successive reduction of available therapeutic options. The emergence and spread of new forms of resistance continues to raise the alarm for this global crisis, especially resistance shared through genetic mobile elements, as antibiotic resistant microbes can transmit their resistance genes, increasing resistance. Due to the extreme gravity of this issue, it is necessary to investigate and understand the new threats and resistance mechanisms of MDR bacteria or fungi. This Research Topic integrates recent studies on the new threat of MDR infection in clinic, novel characteristics and mechanisms of carbapenem-resistant *Enterobacterales* (CRE), as well as surveillance information and control strategies of resistance to new antibiotics.

Currently MDR pathogens constitute the main challenge in patients admitted to ICU, which is largely due to invasive procedures, impaired protective mechanisms, and the extensive use of antibiotics. In our collection, Odih et al., using whole genome sequencing (WGS) and single nucleotide polymorphism (SNP) analysis, reported the first outbreak of a carbapenem-resistant IC2 *A. baumannii* clone in an ICU in Nigeria. They also highlighted the importance of *A. baumannii* detection and prevention in Nigerian clinical settings. In another study from China, Wang et al. focused on evaluating the risk factors of acquired *S. maltophilia* pneumonia in ICU patients. This study concluded that the prevalence of hospital-acquired *S. maltophilia* pneumonia in ICU patients was high, and severe diseases, undergoing invasive procedures, and recent antibiotic use may be important contributors. A third study targeting MDR in the ICU, by Çaglayan et al., proposed a data-driven modeling framework to predict MDR colonization upon ICU admission, and identified the associated socio-demographic and clinical factors. In this large study, MDR colonization was found in 17.59% of 4,670 ICU admissions, and it was associated with long-term care facility stay, underlying diseases, and recent precaution procedures before ICU admission.

Another new MDR threat in our topic have described the emerging infection or coinfection with MDR bacteria and fungi. In a case report, Cui et al. described an acute T lymphoblastic leukemia patient with mixed infections of lethal invasive Mucormycosis and multidrug resistant bacteria. Also, they emphasized the importance of considering malignant hematological conditions and using metagenomic next-generation sequencing (mNGS) to aid in the early and timely diagnosis and treatment of Mucormycosis. Another study conducted by Damas et al. focused on *Chryseobacterium indologenes*, an emerging multidrug resistant nosocomial pathogen, and presented the first detailed molecular characteristics of MDR *C. indologenes* through whole-genome sequencing. The researchers also concluded that their findings could help shape future public health policy and MDR *C. indologenes* infection control. Additionally, insights into the impact of extended-spectrum cephalosporin-resistant (ESC-R) *Enterobacterales* (EB) causing early urinary tract infection (UTI) in kidney transplant recipients, were provided by Aramwittayanukul et al. They determined administration of carbapenem peri-transplant prophylaxis can significantly protect against ESC-R EB UTI early after KT. In addition, Xiang et al. looked into the risk factors and outcomes of postoperative pneumonia. They discovered that postoperative pneumonia was linked to a longer length of hospital stay, a higher ICU occupancy rate, a higher rate of unplanned re-operation, and a higher rate of in-hospital mortality.

Carbapenem-resistant Klebsiella pneumoniae (CRKP), a major threat to global public health, has showed some novel characteristics in our Research Topic. Zeng et al. reports the novel findings on epidemiology and resistance mechanisms in ST 11 carbapenem-resistant *Klebsiella pneumoniae* carry *bla*_KPC − 2._ Interestingly, the whole-genome sequencing showed that *bla*_KPC − 2_ was found in a genetic context with insertion sequences ISKpn27 upstream and ISKpn6 downstream, all of which were flanked by IS26. Furthermore, the structure of the type IV secretion system (T4SS) aids in the adaptation of bacteria to the environment.

To combat MDR pathogens, effective control strategies need to be explored. Aside from rapid diagnosis and new antibacterial development, appropriate precaution and necessary surveillance are critical. The study by Morel et al. determined the essential elements and requirements of antimicrobial resistance surveillance for new antibiotics. Another work by Amin et al. discussed the practical antibiotic stewardship in outpatient settings in the United States, and they suggested that community prescribers can help move the needle on antibiotic stewardship by keeping in mind the “4 Ds”: prescribe an antibiotic for a bacterial infectious Disease, with the appropriate Drug, Dose, and Duration.

In summary, the manuscripts in this Research Topic highlighted new threats and novel mechanisms of multidrug-resistant pathogens broadly. We thank all the editors, authors, reviewers for their contributions to this Research Topic. Looking forward, studies involving the new emergence and mechanisms of MDR fungi are highly encouraged in future collections.

## Author contributions

XJ and SH drafted the manuscript. Y-WT and CC revised and edited the Editorial. All authors approved the final version of the manuscript.

## Conflict of interest

Author Y-WT is employed by Danaher Diagnostic Platform/Cepheid. The remaining authors declare that the research was conducted in the absence of any commercial or financial relationships that could be construed as a potential conflict of interest.

## Publisher's note

All claims expressed in this article are solely those of the authors and do not necessarily represent those of their affiliated organizations, or those of the publisher, the editors and the reviewers. Any product that may be evaluated in this article, or claim that may be made by its manufacturer, is not guaranteed or endorsed by the publisher.

